# Sociodemographic Correlates of Alcohol Abuse in Kassena-Nankana Municipality, Ghana

**DOI:** 10.1155/2020/4375420

**Published:** 2020-11-05

**Authors:** John Nyaaba Anyinzaam-Adolipore, Abdul Rauf Alhassan

**Affiliations:** ^1^Department of Supervision, Inspectorate Unit, Ghana Education Service, P.O. Box KA 20, Karaga, Ghana; ^2^Department of Surgery, Tamale Teaching Hospital, P.O. Box TL 16, Tamale, Ghana

## Abstract

The main aim of the study was to assess the level of alcohol abuse and related factors in Kassena-Nankana Municipal of Ghana. The study was conducted using a cross-sectional survey with 397 participants, using AUDIT to assess alcohol use. Data entry and analysis was done using SPSS. Bivariate analysis was done using chi-square and multivariate analysis was done using the multinomial logistics regression model. Lifetime alcohol use among the study participants was 96.0%; out of this, 51.7% were engaged in possibly dependent drinking, 23.4% involved in harmful drinking, and 24.9% involved in moderate drinking. Males were more likely to engage in harmful drinking than moderate (AOR = 2.4, 95% CI: 1.175–4.776). Males again were more likely to engage in dependent drinking than moderate (AOR = 2.7, 95% CI: 1.489–5.068). Christians as compared to traditionalists were less likely engage in dependent drinking than moderate drinking (AOR = 0.03, 95% CI: 0.223–0.940). Those with tertiary education were less likely to engage in dependent drinking than moderate as compare to those without formal education (AOR = 0.2, 95% CI: 0.076–0.670). Also employed civil servants were more likely to engage in dependent drinking than moderate as compared to those without employment (AOR = 4.4, 95% CI: 1.187–16.646). This study revealed a high prevalence of alcohol abuse among the residents of Kassena-Nankana municipality that was predicted by gender, educational level, and religious practice; therefore, there is a need for a public campaign on the harmful effects of alcohol abuse in the municipality.

## 1. Introduction

Alcohol abuse is one of the prominent risk factors for population health worldwide and has a direct influence on the numerous targets of the Sustainable Development Goals (SDGs) relative to health, which include those for mother and child health, infectious diseases (HIV, viral hepatitis, and tuberculosis), noncommunicable diseases, mental health, injuries, and poisonings [1].

In 2016, alcohol misuse was related to some 3 million deaths (5.3% of all deaths) globally and 132.6 million disability-adjusted life years (DALYs), that is, 5.1% of all DALYs in that year. Deaths related to alcohol misuse are higher than those related to other diseases such as tuberculosis, HIV/AIDS, and diabetes. Among men in 2016, an estimated 2.3 million deaths and 106.5 million DALYs were associated with alcohol use or misuse. Women experienced 0.7 million deaths and 26.1 million DALYs were associated with alcohol use or misuse [1].

It is estimated that 23.3% of the Ghanaians drink alcohol and of these, 2.1% are engaged in heavy drinking with the per capita alcohol consumption being 20 liters a year [2]. Alcohol misuse remains one of the topmost significant public health problems irrespective of the active efforts made to solve it [3]. Alcohol remains one of the first substances that are abused by people before they progress to the use of more hazardous substances such as marijuana and cocaine [4]. With modernization in the world market and an increase in advertising, harmful drinks that were not easily accessible to the public are now more often consumed [5]. A survey conducted in the United States of America indicated that the average age of initiation to alcohol use among young people is 15 [6]. Osei-Bonsu et al. found that the prevalence of alcohol use among the youth alone was estimated to be around 43%. Both males and females used alcohol, but more males were reported to use alcohol than females and the major factors influencing use were peer influence and alcoholic drinks advertising [4].

Data from the Ghana Demographic and Health Survey found alcohol use to be common in Ghana with Volta Region recording the highest prevalence for males at about 42%, while among females the highest prevalence of alcohol use was in the Upper West Region at 37% [7]. Despite the generally high rates of alcohol consumption, the pattern varies by country and even different regions of countries. Alcohol consumption and related patterns vary from urban to rural areas and also according to the level of wealth. The rates of mortality and morbidity per capita alcohol consumption are also high for low-income countries as compared to high-income countries [1].

Sandema is a town 15 km away from Kassena-Nankana. According to the Paramount Chief of Sandema, alcohol misuse is destroying many people in the Traditional Area and urged authorities of the municipality to impose heavy levies on alcoholic beverages brought to the area, especially Akpeteshie, to make them expensive and discourage the people from abuse [8]. Alcohol comes in the form of beer, wine, spirit, and other alcoholic beverages.

In the absence of a fully functional national policy coupled with inadequate documentation on alcohol-related harm or deaths in the Kassena-Nankana municipality, it is feared that there may be a public health emergency which is yet to be recognized; hence, this study aimed to assess the alcohol abuse level and factors associated with this abuse in Kassena-Nankana municipality.

## 2. Aim of the Study

The main aim of the study was to assess the alcohol consumption level among the residents of Kassena-Nankana municipality and the factors associated with the consumption level.

## 3. Methods

This study was conducted using a descriptive cross-sectional survey. The study population was all people aged 15–65 years residing in the Kassena-Nankana Municipality at the time of the study. This age group was chosen because the previous and current studies report that alcohol intake is high within these age brackets.

### 3.1. Sampling and Sample Size Determination

The sample size was calculated using the Yamane formula for calculating sample size which is stated below, where *n* is the sample size, *N* is the population size, and *e* is the level of precision (*N* = 109944, *e* = 0.05):

(1) $$\begin{array}{c} n=\frac{N}{1+N{\left(e\right)}^{2}}\;n=\frac{109944}{1+109944{\left(0.05\right)}^{2}}=399. \end{array}$$

For nonresponse, 5% of the 399 was added to get the final sample of 419.

A multistage sampling technique was used for this study; a simple random sampling technique was used to select the first house. A systematic sampling technique was used to select every 27^th^ house from the 11503 houses in the municipality. Simple random sampling was used to select respondents (15–65 years) from the houses.

The alcohol use disorder identification test (AUDIT) was also adopted to assess the level of alcohol use in the municipality. The interviews were conducted by researchers plus trained research assistants in the homes of the participants. The interviews were done in English with the help of translators where needed.

### 3.2. Method of Data Analysis

Data entry and analysis were done using Statistical Package for the Social Sciences (SPSS) version 20. Continuous variables were summarized into means and proportions, while categorical variables were summarized into frequencies and percentages and presented using tables. Chi-square analysis was used for bivariate analysis and multinomial regression for multivariate analysis.

Every question on alcohol AUDIT test scores varies from 0 to 4, with the first response for every question score of 0 for never, the second (e.g., less than monthly) for score 1, the third (e.g., monthly) for score 2, the fourth (e.g., weekly) for score 3, and the last response (e.g., daily or almost daily) scoring. For questions 9 and 10, which only have three responses, the scoring is 0, 2, and 4 (from left to right). A score of 8 or more suggests harmful or hazardous drinking, a score of 13 or more in women and 15 or more in men is suggestive of alcohol dependence [9].

### 3.3. Ethical Consideration

The researchers sought approval from the Kassena-Nankana Municipal Assembly, Kassena-Nankana Municipal health directorate, and the Naranjo Health Research Centre. Respondents' consented to participate in the study and they were made to know that they had the right to skip any question they felt uncomfortable answering and could withdraw from participating at any time. Confidentiality was assured and any form of harm avoided.

## 4. Results

### 4.1. Demographic Characteristics of Participants

Out of the 419 questionnaires used, 397 were correctly responded to, representing a response rate of 94.7%. About 56.7% of them were males, 36.8% of them had no formal education and many (71.0%) were unemployed. Two hundred and fifty-six (64.5%) of the participants were married and about 60.2% of the participants were Christians (Table 1).

### 4.2. The Prevalence of Alcohol Use in the Municipality

Question one on the alcohol AUDIT test questionnaire (How often do you have a drink containing alcohol?) was used to classify prevalence. Those with a history of ever using alcohol were classified and, out of the 397 participants, 381 (96.0%) have a history of alcohol use and to measure the extent of alcohol abuse and associated factors, those (4.0%) without alcohol use history were excluded from completing the remaining questions on the alcohol AUDIT test questionnaire and not included in subsequent analysis (Table 2).

On the participant choice of drink, most (37.8%) the participants preferred beer, followed by 22.0% who preferred spirits and then 19.4% who preferred other local alcoholic drinks such as “fighter,” “Kpookeke,” “Odo Nsa,” “Round 2,” and “Kpaalongo bitters”; also 15.5% of them preferred Guinness and least (5.3%) preferred paper wine (a local sachet wine make from cassava).

### 4.3. Alcohol AUDIT Test and Classification of Alcohol Abuse

The mean score of all (381) the participants was 15.7 ± 10.2, the minimum score was 1, the maximum score was 40, and the modal score was 10.0. A score of 8 or more is associated with harmful or hazardous drinking; a score of 13 or more in women and 15 or more in men is likely to indicate alcohol dependence. The majority (51.7%) of the study participants are scored at the level suggestive of dependence, 24.9% of the study participants were engaged in low risk or moderate drinking, and 23.4% of them were engaged in harmful drinking. Table 2 represents the questions on the AUDIT test (AUDIT 1–10) and various responses from study participants.

### 4.4. Relationship between Level of Alcohol Use and Independent Factors

From the chi-square analysis only two of the studied independent variables were not statistically related to the level of alcohol use or abuse. These were age and marital status (*P* > 0.05). The remaining studied variables were significant: sex (*X*^2^ (2, 381) = 8.526, *p*=0.014); educational level (*X*^2^ (8, 381) = 21.751, *P*=0.05); occupation (*X*^2^ (6, 381) = 14.223, *P*=0.027P); religious affiliation (*X*^2^ (4, 381) = 25.095, *P* ≤ 0.001); and respondents' choice of alcoholic drink (*X*^2^ (8, 381) = 19.182, *P*=0.014) (Table 3).

### 4.5. Multinomial Logistics Regression Model for the Level of Alcohol Use and Associated Variables

Independent variables that were significant in bivariate analysis were further modeled to identify predictor variables using multinomial logistical regression. In the model comparing harmful drinking to moderate drinking, the males were 2.4 times more likely to engage in harmful drinking than moderate as compared to females (AOR = 2.4, 95% CI: 1.175–4.776) (Table 4).

Also, comparing dependent drinking to moderate drinking, males again were 2.7 times more likely to engage in dependent drinking than moderate as compared to females (AOR = 2.7, 95% CI: 1.489–5.068). Regarding religious affiliation, Christians were 50% less likely to engage in dependent drinking than moderate drinking as compared to traditionalist (AOR = 0.5, 95% CI: 0.223–0.940). Those with tertiary education were 20% less likely to engage in dependent drinking than moderate as compared to those without formal education (AOR = 0.2, 95% CI: 0.076–0.670). Also employed civil servants were 4.4 times less likely to engage in dependent drinking than moderate as compared to those without employment (AOR = 4.4, 95% CI: 1.187–16.646) (Table 5). The data of the study fits the model, given that the Goodness-of-Fit result is not statistically significant (*X*^2^ = 267.040, *P*=0.095).

## 5. Discussion

The study found a very high prevalence of alcohol use (96.0%). This is higher than the national prevalence of 55.5% in 2004 and 30.5% according to WHO Global Status Reports on Alcohol and also higher than that of the general population in Sub-Saharan Africa which stands at 60% according to the World Health Organization [1]. Though, the methods of data collection were similar (surveys), WHO studies were nationwide surveys and this current study was a survey of one municipality and this could account for variation in the demographic structures of the two populations. Moreover, demographic variations such as religious affiliation may account for the large difference in alcohol use prevalence. Also, the time of the data collection could be a contributing factor because data were collected from December 2018 to January 2019 during the Christmas season, which makes it more likely that participants had consumed alcohol recently. Beer was found to be the most common alcoholic drink of choice (37.8%), followed by spirits and others (18.89%). This is different from an earlier finding by the World Health organization that globally spirits are the most consumed alcohol of choice [1].

Although there was no significant relationship between age and AUDIT scores, young people between the ages of 15 and 29 years (31.0%) were alcohol consumers. Some of them were Junior High School graduates as well as Senior High School graduates. This is consistent with earlier findings by the Ghana Demographic and health survey which reported the prevalence of alcohol use among age groups of 15–34 years [7]. The finding of this study is also consistent with the findings from the Upper West Region of Ghana, in which the authors found in their study that by the age of 17 years many participants were lifetime drinkers [10]. However, the majority of respondents (49.9%) in this study were between the ages of 30 and 49 years. This is the age when alcohol drinking peaks according to the Ghana Statistical Service report in 2008 [7].

Marital status was not related to AUDIT scores, which is also inconsistent with a study finding that divorce was related to increased heavy drinking, with noticeable short-term effects [11].

Gender was strongly associated with AUDIT scores. This is consistent with the finding of the Ghana Statistical Service, reporting the prevalence of alcohol drinking among males to be 36% and that of the females to be 17.5% [7], and it is also congruent with the general finding that alcohol-use disorders are more prevalent in men than women [12].

The current study also found that there was an association between education and severity of AUDIT scores. Those with tertiary education were likely 20% more likely to meet the cutoff suggesting dependence, compared to those without formal education. This differs from the Ghana Statistical Services survey which found that there were no differences in alcohol use by demographic characteristics such as level of education, wealth quartiles, and urban-rural residence [7]. But, the finding is consistent with the Anyawie study, which found that more educated people tend to drink less than less educated people [13].

The occupation of the participant was also related to the severity of AUDIT scores. Civil servants were 4.4 times more likely to engage in dependent drinking, relative to moderate drinking, compared to those who were not employed. This view is consistent with the previous findings that affluent people are more likely to drink more alcohol than less affluent ones and more likely to engage in hazardous drinking [13, 14]. It is also consistent with the findings by the Ghana Statistical Service that found that the prevalence of alcohol consumption was higher among women who were employed than those who were unemployed [7].

The study found a relationship between religious affiliation and alcohol use or abuse. Christians were 50% less likely to engage in dependent drinking relative to moderate drinking, compared to traditionalists, similar to that which has been reported previously [15].

Finally, a preference for beer was associated with higher AUDIT scores, which is similar to a study that revealed beer as a choice of an alcoholic drink predicted alcohol abuse [16].

This study was not without limitations since the questions asked on alcohol ever use and quantity or intensity of use depended on memory; therefore, there is the possibility of recall bias.

To sum up, this study found very high AUDIT scores, with over half of participants scoring higher than the standard cut-off suggestive of alcohol dependence. Higher scores were associated with a preference for beer, male gender, affluence, as measured by occupation and education, and Christian religious affiliation. There is a need for a public campaign on the harmful effects of alcohol abuse in the municipality. There is also a need for media advertisements for alcoholic beverages to be regulated by the national media commission. To address the harmful use of alcohol, the government and society need to be involved, with proper engagement of public health-oriented NGOs, professional associations, and civil society groups.

## 6. Conclusion

The study revealed very high alcohol AUDIT scores among the participants, which was associated with socioeconomic factors such as sex of the participant, religious affiliation, level of education, and employment status.

## Figures and Tables

**Table 1:** Demographic characteristics of participants.

		Frequency (*n* = 397)	Percentage
Sex	Male	225	56.7
	Female	172	43.3

Age	15–29	124	31.2
	30–49	194	48.9
	50–65	79	19.9

Education level	Primary	49	12.3
	JHS	55	13.9
	SHS	61	15.4
	Tertiary	86	21.7
	No formal education	146	36.8

Occupation	Artisan	28	7.1
	Public servant	58	14.6
	Civil servant	29	7.3
	Other-specify	281	71.0

Marital status	Married	256	64.5
	Single	88	22.2
	Widowed	41	10.3
	Divorced	12	3.0

Religious affiliation	Christian	239	60.2
	Muslim	23	5.8
	Traditionalist	135	34.0

**Table 2:** Participants' responses to the questions of the alcohol AUDIT questionnaire.

	Alcohol AUDIT score					Total
	0	1	2	3	4	
How often do you have a drink containing alcohol?	16	91	66	94	130	397
How many standard drinks containing alcohol do you have on a typical day when drinking?	173	100	45	27	36	381
How often do you have six or more drinks on one occasion?	207	36	50	38	50	381
During the past year, how often have you found that you were not able to stop drinking once you had started?	134	25	54	37	131	381
During the past year, how often have you failed to do what was normally expected of you because of drinking?	171	34	64	56	56	381
During the past year, how often have you needed a drink in the morning to get yourself going after a heavy drinking session?	232	19	29	27	74	381
During the past year, how often have you had a feeling of guilt or remorse after drinking?	170	26	66	52	67	381
During the past year, have you been unable to remember what happened the night before because you had been drinking?	208	26	52	45	50	381
Have you or someone else been injured as a result of your drinking?	227	0	69	0	85	381
Has a relative or friend, doctor, or other health worker been concerned about your drinking or suggested you cut down?	118	0	124	0	139	381

The prevalence of alcohol ever use is never use (16) deducted from total respondents (397) divided by 397 multiple 100.

**Table 3:** Chi-square analysis for the relationship between the level of alcohol use and independents factors.

		Level of alcohol use			df	*X* ^2^	*p*-value
		Moderate	Harmful	Defendant			
Age	15–29	38	24	56	4	6.619	0.157
	30–49	40	51	99	
	50–65	17	14	42	

Sex	Male	42	53	122	**2**	**8.526**	**0.014** ^*∗*^
	Female	53	36	75			

Education level	Primary	11	6	27	**8**	**21.751**	**0.005** ^*∗*^
	JHS	8	20	25	
	SHS	16	14	29	
	Tertiary	31	20	31	
	No formal education	29	29	85	

Occupation	Artisan	6	10	11	**6**	**14.223**	**0.027** ^*∗*^
	Public servant	21	13	20	
	Civil servant	4	4	20	
	Unemployed	64	61	146	

Marital status	Married	59	57	130	6	5.743	0.453
	Single	28	18	39	
	Widowed	7	11	21	
	Divorced	1	3	7	

Religious affiliation	Christian	74	60	97	**4**	**25.095**	**0.001** ^*∗*^
	Muslim	3	5	11	
	Traditionalist	18	24	89	
	Other-specify	0	0	0	

What is your alcohol of choice	Beer	42	44	58	**8**	**19.182**	**0.014** ^*∗*^
	Guinness	17	15	27	
	Paper wine	5	3	12	
	Spirits	14	14	56	
	Other local drinks	17	13	44	

^*∗*^The Chi-square statistic is significant at the 0.05 level.

**Table 4:** Multinomial logistics regression model for the level of alcohol use and independent variables (first coefficient table).

Level of alcohol use		*B*	Wald	Sig.	AOR	95% confidence interval for AOR	
						Lower bound	Upper bound
Harmful drinking/moderate drinking	Intercept	−0.299	0.399	0.528	**2.369**	1.175	4.776
	Male	0.862	5.811	**0.016**			
	Female	0^b^	—	—			
	Christian	−0.364	0.703	0.402	0.695	0.297	1.627
	Muslim	0.502	0.324	0.569	1.652	0.293	9.305
	Traditionalist	0^b^	—	—	—	—	—
	Primary	−0.771	1.699	0.192	0.462	0.145	1.475
	JHS	0.718	1.771	0.183	2.050	0.712	5.900
	SHS	−0.430	0.660	0.417	0.650	0.230	1.837
	Tertiary	−0.703	1.319	0.251	0.495	0.149	1.644
	No formal education	0^b^	—	—	—	—	—
	Artisan	0.687	1.437	0.231	1.987	0.647	6.104
	Public servant	−0.175	0.095	0.758	0.839	0.275	2.558
	Civil servant	0.148	0.034	0.853	1.160	0.241	5.576
	Unemployed	0^b^	—	—	—	—	—
	Beer	0.397	0.714	0.398	1.487	0.592	3.732
	Guinness	0.149	0.071	0.789	1.161	0.389	3.465
	Paper wine	−0.283	0.105	0.746	0.754	0.136	4.182
	Spirit	0.136	0.061	0.805	1.146	0.388	3.386
	Other local drinks	0^b^	—	—	—	—	—

The reference category is a moderate drinker.

**Table 5:** Multinomial logistics regression model for the level of alcohol use and independent variables (second coefficient table).

Level of alcohol use		*B*	Wald	Sig.	AOR	95% Confidence interval for AOR	
						Lower bound	Upper bound
Dependent drinking/moderate dinking	Intercept	1.184	9.624	0.002	**2.747**	1.489	5.068
	Male	1.010	10.453	**0.001**			
	Female	0^b^	—	—			
	Christian	−0.780	4.532	**0.033**	**0.458**	0.223	0.940
	Muslim	0.502	0.397	0.529	1.652	0.347	7.874
	Traditionalist	0^b^	—	—	—	—	—
	Primary	−0.233	0.261	0.609	0.793	0.325	1.933
	JHS	0.060	0.014	0.905	1.062	0.396	2.847
	SHS	−0.547	1.376	0.241	0.579	0.232	1.443
	Tertiary	−1.491	7.181	**0.007**	**0.225**	0.076	0.670
	No formal education	0^b^	—	—	—	—	—
	Artisan	−0.051	0.008	0.927	0.950	0.317	2.849
	Public servant	0.338	0.423	0.515	1.402	0.507	3.876
	Civil servant	1.492	4.905	**0.027**	**4.445**	1.187	16.646
	Unemployed	0^b^	—	—	—	—	—
	Beer	−0.483	1.459	0.227	0.617	0.281	1.351
	Guinness	−0.307	0.428	0.513	0.736	0.294	1.845
	Paper wine	0.160	0.056	0.813	1.174	0.312	4.418
	Spirit	0.149	0.112	0.738	1.161	0.484	2.785
	Other local drinks	0^b^	—	—	—	—	—

The reference category is a moderate drinker.

## Data Availability

The primary data related to the findings of this study are available from the corresponding author upon request.
